# Biodegradable Packaging Materials from Animal Processing Co-Products and Wastes: An Overview

**DOI:** 10.3390/polym13152561

**Published:** 2021-07-31

**Authors:** Diako Khodaei, Carlos Álvarez, Anne Maria Mullen

**Affiliations:** Department of Food Quality and Sensory Science, Teagasc Food Research Centre, Ashtown, Dublin, Ireland; diako.khodaei@teagasc.ie (D.K.); carlos.alvarez@teagasc.ie (C.Á.)

**Keywords:** biodegradable polymers, packaging materials, meat co-products, animal by-products, protein films

## Abstract

Biodegradable polymers are non-toxic, environmentally friendly biopolymers with considerable mechanical and barrier properties that can be degraded in industrial or home composting conditions. These biopolymers can be generated from sustainable natural sources or from the agricultural and animal processing co-products and wastes. Animals processing co-products are low value, underutilized, non-meat components that are generally generated from meat processing or slaughterhouse such as hide, blood, some offal etc. These are often converted into low-value products such as animal feed or in some cases disposed of as waste. Collagen, gelatin, keratin, myofibrillar proteins, and chitosan are the major value-added biopolymers obtained from the processing of animal’s products. While these have many applications in food and pharmaceutical industries, a significant amount is underutilized and therefore hold potential for use in the generation of bioplastics. This review summarizes the research progress on the utilization of meat processing co-products to fabricate biodegradable polymers with the main focus on food industry applications. In addition, the factors affecting the application of biodegradable polymers in the packaging sector, their current industrial status, and regulations are also discussed.

## 1. Introduction

Plastics play an important role in our world and naturally occurring polymers such as rubber, waxes, resins, and horn have been used in for a variety of applications since ancient times. However, since the 19th century and with the development of petroleum-based thermoplastics, a revolution in plastic industry has occurred [1]. Petroleum-based plastics, due to their light weights, low production costs, durability, resistance against corrosions, thermal, and electrical insolation, have gained interest for a wide variety of applications from technological advances to the packaging sector [2]. However, the increasing generation and usage of petroleum-based materials in the recent decades, has put an immense stress on the environment through generation of plastic waste and their accumulation in landfills [3] and oceans [4]. Nearly 50 percent of the plastics are employed in single-use applications, such as disposable consumer items and packaging, with food packaging accounting for the majority of plastic waste [5]. Many of these plastics are used in plastic bags or on food products, with a lifetime of mere minutes to hours, while the plastics stay in the environment for decades. It is estimated that the oceans are polluted with approximately 100–200 million tons of plastic waste, with 8 million tons entering the waters each year [6]. Micro-plastics generated from plastics breakdown are reaching alarming levels in the air, water, seafood, and table salt presenting a serious health issue for marine wildlife and humans [7,8]. Although only a low percentage of the carbon footprint is attributed to fossil-based plastic materials (4% of European greenhouse gas emissions are from using plastics); looking for alternative packaging materials with a lower carbon footprint and less emission of greenhouse gases is environmentally and economically interesting, and also helps the sustainability of the value chain as outlined in the “European Strategy for Plastics in a Circular Economy” released in 2018. Plastic packaging materials are one of the major challenges for waste management and the environment. For example, less than 30% of plastic waste generated in Europe is recovered for recycling and the majority is exported to the Southeast Asia [9].

**Figure 1:**
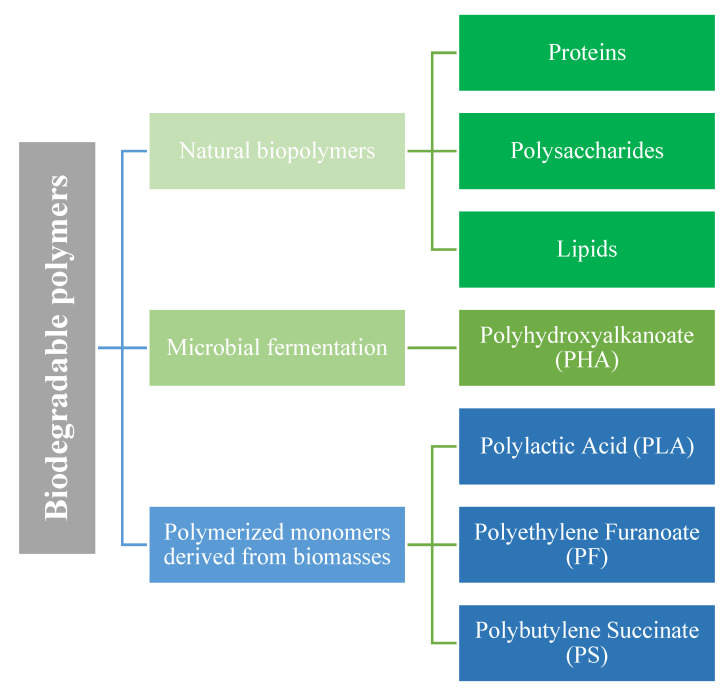
Biodegradable polymers from different sources.

By acknowledging the serious environmental and health issues attributed to petroleum plastics, and the inevitable demand for their application in various industries, research in the development of environmental-friendly plastics has gained huge interest in the past decades [10,11,12]. Moreover, the European Union Sustainable Development Strategy [13] and the United Nations 2030 Sustainable Development Goals [14], particularly in the packaging supply chain, have accelerated the demand for shifting from petroleum-based packaging to the bio-based plastics that are more effective, safer for human, and more eco-friendly [15]. Bioplastic materials are commonly categorized in two major groups: the first group is recyclable polymers, which are partially or completely made from biological and sustainable sources such as grains, starchy root vegetables, sugar cane or vegetable oils. The second group is biodegradable plastics that can be degraded by natural microorganisms present in soil or water into carbon dioxide (CO_2_), methane (CH_4_), water, and inorganic compounds [16]. The life cycle for biodegradable packaging materials is shown in Figure 1.

### 1.1. Biodegradable Packaging Materials

**Figure 2:**
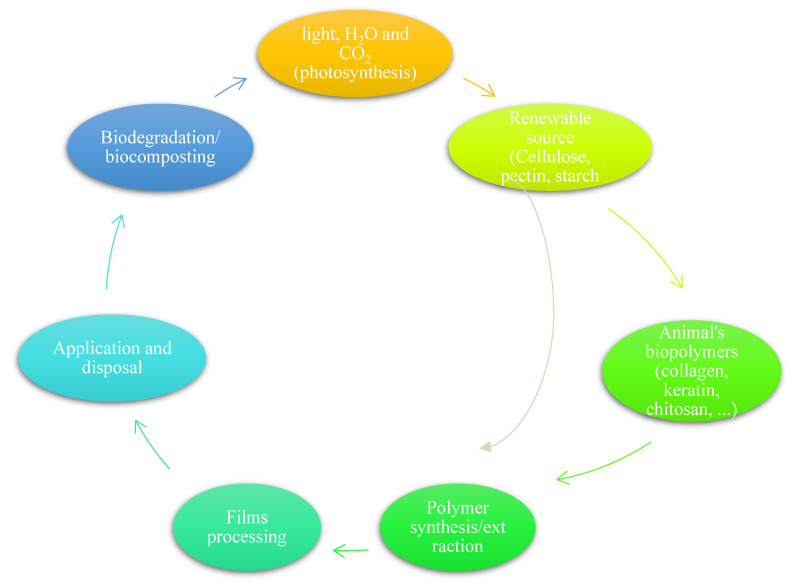
Life cycle for biodegradable polymers from natural sources [22].

Biodegradable packaging materials are commonly generated from sustainable natural resources or from the by-products of food and agricultural products and, based on the raw material used, can be categorized into three main groups (Figure 2). These non-toxic polymers exhibit considerable mechanical and barrier properties, and can be biodegradable and compostable making them excellent candidate material for food and agricultural applications [17]. Polylactic acid (PLA), an aliphatic polyester from lactic acid or lactide monomers, is one of the most studied and prevalent biodegradable polymers with various applications in the industrial packaging sector. PLA is manufactured by the controlled fermentation of carbohydrates from different bio-sources such as corn starch or sugarcane. PLA films have a good mechanical and barrier properties but due to the slow degradation rate in mild home composting conditions and their high dependency on hydrolysis for complete degradation, they need industrial composting conditions to be degraded [18]. Moreover, PLA production is associated with a high water footprint (0.248 m^3^/kg) mainly due to maize cultivation [19]. Utilization of edible and nutritive plants such as corn, cassava, sugarcane, and sugar beet pulp are another drawback attributed to the industrial production of PLA. Therefore, recycling PLA into LA monomers is preferable from an energy point of view rather than producing LA from glucose fermentation [20]. Polyhydroxyalkanoate (PHA) is another bio-based polymer with good mechanical and barrier properties which is completely biodegradable. PHA is manufactured from microbial fermentation of carbon sources and nutrients under nutritional stress [21]. Nevertheless, the high price of manufacturing PHA is limiting its application in the packaging sector, therefore, it is mainly used in high added-value products in pharmaceutical or medical applications [21].

Natural biopolymers, such as polysaccharides, proteins, and lipids, can be found abundantly from plant and animal sources and they have great potential to be used as feedstock for biodegradable packaging materials. Polysaccharides are the most abundant natural polymers in nature. Starch, cellulose, and its derivatives, pectin, chitosan, alginate, carrageenan, pullulan, and gellan gum are some of the polysaccharides that have been extensively studied for producing biodegradable films. Polysaccharide films are associated with a low toxicity, mechanical stability, oil and lipids barrier properties, and selective permeability against oxygen transfer. Application of polysaccharides in edible films and coatings has been extensively reviewed elsewhere [23]. A characteristic of these films is that they are commonly brittle and hydrophilic, which reduces their attractiveness for food packaging. The source of polysaccharide, the degree of substitution in cellulose, the degree of modification in starch, chemical groups attached to monosaccharides, and the molecular weight of polysaccharides are the most important factors influencing the properties of these films.

Proteins possess good film-forming properties mainly due to their ability to form an amorphous three-dimensional framework primarily stabilized by non-covalent interactions. The most common proteins used for films production are collagen, gelatin, caseins, whey proteins, myofibrillar proteins, quinoa protein, soy protein, and keratin [24]. Physicochemical properties of protein films are influenced by several factors including protein source (plant or animal based) and purity, amino acid composition, denaturation degree, pH, and the presence of additives. Generally, protein-based films have stronger mechanical, optical, and barrier properties compared to polysaccharide-based films [25]. Electrostatic interactions, hydrogen bonds, ionic bonds, Van der walls forces, covalent bonding, hydrophobic bonds, and disulfide bridges are the many bonds and interactions which are involved in stabilizing protein films. One of the major drawbacks of protein-based films, also the case for those produced from polysaccharides, is the negative impact of moisture on the functional properties of the films due to their hydrophilic nature [26,27].

Lipid-based edible films and coatings are efficient barriers against moisture transfer due to their nonpolar nature [28]. Plant and animal waxes, vegetable oils, and fatty acids are the most popular lipids used for preparing film forming solutions [29]. Brittle structure of the lipid-based films limits their application when used alone; therefore, they are often used in combination with hydrophilic hydrocolloids in emulsion or bilayer form [30,31]. Cellulose ethers, pectin, chitosan, starch, alginates, and carrageenan are the most cited hydrocolloids used to form composite films [32] that can be often combined with stearic or palmitic acids, beeswax, acetylated monoglycerides, and lecithin [33].

### 1.2. Important Parameters to Be Considered in Biodegradable Films Production

One of the main purposes of packaging is to protect the product, in terms of quality and safety, during transportation and storage [34,35]. Packaging materials, used for food packaging, are required to possess a low permeability to water and exhibit acceptable mechanical strength and flexibility. However, biodegradable films obtained from natural polymers tend to have lower mechanical and barrier properties compared to those produced using synthetic polymers, thus limiting their industrial applications [36]. Protein and polysaccharide films, due to their hydrophilic nature, exhibit a higher permeability to water than synthetic films and generally have weaker and less flexible structure. The characteristics of the polymers, employed as building blocks, dictate their potential application in packaging sector, as different products demand specific properties to guarantee their safety. Mechanical properties, barrier characterization, color and optical properties, water solubility, biodegradability, and sealing properties are the main criteria of packaging polymers in industrial production.

Mechanical properties, such as tensile strength and flexibility, are important attributes of flexible packaging materials, influencing the barrier properties, integrity and product’s attractiveness. These are commonly influenced by the source of the biopolymer, manufacturing methods, thickness of the film, and the relative humidity of the surrounding. One of the major drawbacks of bio-based films is their weaker mechanical properties compared to the synthetic polymers. Incorporation of plasticizers such as glycerol, sorbitol, or poly-ethylene glycol, are suggested to increase the mobility of these biopolymer chains by positioning between their molecules [37]. Chemical modifications, enzymatic cross-linking, physical treatments (irradiation or heat treatment), incorporation of different compounds such as essential oils or nanoparticles are other proposed methods to improve the mechanical strength and flexibility of biodegradable films [38].

Water barrier and gas barrier properties are important parameters for evaluation of food packaging materials as they can give information about the chemical structure, free volume between molecules in the film matrix, water affinity, crystallinity, and cross-linking of polymers. Mechanical and barrier properties of bio-based polymers are directly linked to their microstructure properties [34]. Polysaccharide and protein films are generally excellent gas barriers with a high tendency for water vapor permeability. Chemical and enzymatic cross-linking, irradiation, combining with different polymers, and incorporation of nanoparticles are the suggested methods to improve the water barrier properties of biodegradable films. Due to the relatively high water vapor permeability of natural biodegradable polymers, such as proteins and polysaccharides, they are best used for short-term applications as moisture barriers, or can be useful in other applications such as modified atmospheric packaging (MAP) of fruits, vegetables, dairy products like cheese, and fermented foods like fish and meat where high water vapor permeability is required [39].

Color and optical properties of packaging films directly affect the consumer’s preferences, and can also help to protect the product against UV rays [10]. Generally, consumers prefer packaging with a higher transparency and less color alteration as it gives a more realistic view of the product. However, some products are sensitive to the light and require less transparent packaging to visible light and UV in order to prevent oxidation reactions [34].

Water solubility is an important factor influencing the integrity of the packaging material which has been in contact with water and that has a direct effect on food quality. In general, biopolymers have a high solubility in water restricting their application in packaging, especially for products with a high moisture content. Chemical structure modifications or increasing the surface hydrophobicity of bio-based films, are the suggested methods to reduce water solubility of these bio-polymers.

Biodegradation is the change observed in polymeric materials by breaking down the structure and converting them into carbon dioxide, water, methane, inorganic materials, and biomass during contact with enzymes, or microorganisms such as bacteria, fungi, and/or algae. Moreover, biodegradability is an important property for some applications such as biomedical materials. Ideally the generation of such materials should have a limited impact on environment is demanded. According to the European standard EN 13432, biodegradable packaging materials must be disintegrated by at least 90%, via biological activities, in a period of 6 months [36]. The environmental conditions such as temperature, humidity, microbial load, pH, and oxygen content are influencing factors on the biodegradation rate of biopolymers. These natural polymers are generally degraded in industrial composting or home composting conditions depending on the nature of polymer. Therefore, the dumping of biodegradable polymers in the environment without considering their source or method of production might be hazardous to the environment. Thus, it is essential when you consider the chance of accidental dumping or lack of collecting organizations in less developed countries that these materials degrade in a reasonable time period, without releasing dangerous materials and products into the environment. Some polymers, such as PLA, PHA, PBS (polybutylene succinate), or PEF (polyethylene 2,5-furandicarboxylate), require industrial composting as they demand a high temperature (over 50 °C) for initiating hydrolysis and the degradation process. For home composting materials, the biopolymer should exhibit considerable biodegradation and decomposition at ambient temperature. Biodegradable films from proteins, polysaccharides, and waxes are completely biodegradable at home-composting conditions. If the final packaging material is composed of edible films or coating, the risk of dumping such material into the environment is lessened. However, some starch and cellulose derivatives due to the degree of modifications must be degraded under industrial composting condition [21]. The toxicological analysis of intermediate and final products during the degradation assays of biopolymers in the environment is an important factor that should be consideration of biodegradable polymers. Moreover, the life cycle assessment (LCA) of biopolymers is an additional factor to be evaluated to help elucidate the environmental impact of biodegradable materials starting from their raw material extraction to the ultimate application and finally depletion in the environment.

For successful application in industrial packaging the film must demonstrate good sealing properties. This can be limited due to the lower strength and elastic properties of the biodegradable films, compared to petroleum-based plastics [40]. Thermal sealing is the most commonly used technique in the packaging industry with the melted polymers forming a firm seal due to the inter-diffusion and entanglement of polymers from both layers [41]. Use of natural organic adhesives such as starch or dextrin, sugar-based glues, or gelatin solutions are also proposed for non-heat sealing of biodegradable and edible films.

### 1.3. Industrial and Market Status of Biodegradable Plastics

**Figure 3:**
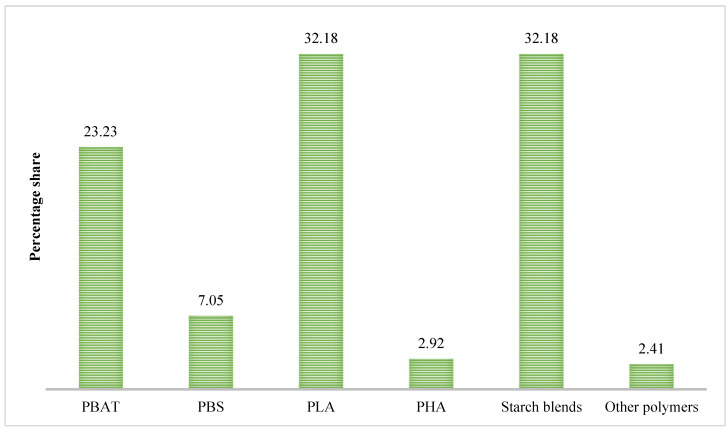
Global production share (%) of biodegradable polymers in 2020 [43].

The total world production of biodegradable plastics in 2019 was 1.17 million tons (58.1% of total bioplastics production) and it is expected to reach to 1.33 million tons by 2024 (European bioplastics, 2020). PLA (32.18%), starch blends (32.18%), and PBAT (Polybutylene adipate terephthalate) (23.23%) are the major biodegradable polymers used in bioplastic production (Figure 3). Key factors influencing this growth are regulatory directives (Section 1.4), increased public awareness concerning the environmental effects, and the increased demand from food packaging, agricultural, and horticultural industries [42]. However, currently the global market for bioplastics (including degradable and non-degradable) only represents one percent of the total plastics produced annually. PLA, with the highest share of the biodegradable films market in 2020, is a widely used polymer in a variety of thermoplastic products such as cups, containers, planter boxes, and takeaway food trays. PHAs, with the current share in total bioplastics production of 1.7% in 2019, showed the highest relative growth rate in production quantities.

In terms of global bioplastics production in 2019, Asian countries accounted for production of 45% of total bioplastics, followed by Europe (25%), North America (18%), and South America (12%). European countries represented the highest level of research and development output in the field of bioplastics as well as industrial development [43]. The packaging sector is a major field for the application for bioplastics (54% of total bioplastics market), followed by textiles, consumers goods, and agriculture and horticulture products. It is estimated that food packaging is the fastest growing sector for application of biodegradable films, which is attributed to the higher demand for this material in fast food, ready to eat meals, fresh and frozen food, dried snacks and candy, and bakery products [44].

Currently, one of the main obstacles to industrial production of bioplastics is the higher production cost when compared to petroleum polymers [45]. For instance, the production cost for PHA is 5–10 times higher than that of petroleum-based polymers [46]. It is considered that the total cost of packaging must be less than 10% of product cost [47], to ensure viability. Considering current industrial practice, the production cost of biodegradable packaging materials must become lower or equal to the petroleum-based films, while maintaining comparable barrier and mechanical properties [48]. Alternatively, a change in the pricing structures is required to accommodate the higher cost. Considering the regulatory changes which are pushing away from reliance on petroleum-based plastics some change is inevitable. Valorization of organic wastes or food industry co-products offers potential for reducing reliance on petroleum-based materials and may also aid in reducing the manufacturing cost of bio-based polymers [15,49]. Conversion of renewable sources into bioplastics, not only helps to migrate into a more circular bio-economy, but also assists in reducing gas emissions, pollution, preserving the ecosystem and biodiversity [50]. However, should such materials not be disposed of in the appropriate manner, their waste disposal will be treated like any other non-degradable plastics, thus not contributing to the circular economy. Proteins, polysaccharides, and lipids from plant and animal sources are excellent renewable sources for developing biodegradable packaging materials, however as outlined earlier further research and innovations are necessary to bring them through to industrial applications.

### 1.4. Regulatory Status

The Food and Drug Administration (FDA) regulates the legislation of food packaging and food contact materials in the USA, to ensure the packaging materials are safe for food contact under conditions of the intended use. The European Union (EU) has also established the regulatory policy for analysis and quality control of food packaging materials since the 1970s in order to regulate both for consumer health and for commercial reasons. The Australian standard applied for food contact materials is AS 2070:1999 which refers to both US FDA 21 CFR and EU FCM regulations. The hygienic standard for use of additives in food containers and packaging materials in China is GB 9685-2008 [51]. To work toward internationally recognized standards, codes of practice, guidelines etc., both United Nations (FAO) and the World Health Organization (WHO) have created the *Codex Alimentarius* [52]. In general, bio-based plastics are required to meet the same regulations with respect to food safety as fossil-based plastics. To access EU markets, additional assessments are necessary for biodegradable polymers to certify their biodegradability or compostability. Health risks assessments and the migration tests (EU No. 10/2011) are other factors that should be included for food contact materials. The migration test is necessary to show the material does not transfer toxic substances to the product and is safe to use for food packaging or agricultural products. Industrial compostable packaging materials are required to be disintegrated by at least 90% after 3 months and their possible eco-toxicity and heavy meal content should be considered (EU standard EN 13432). Although there are no international standards related to the conditions of home composting of biodegradable materials, Australian norm AS 5810 and French standard NF T 51-800 for biodegradable home composting specify that these plastics should degrade at least 90% after 12 months at ambient temperature. According to the EU standard EN 17033, biodegradable mulch films used in agriculture and horticulture, which are manufactured from thermoplastic materials, should be degraded by at least 90% for 48 months at a temperature of 25 °C. Moreover, these materials must meet with SVHC (substances of very high concern) rules and have no negative effects on natural microorganisms from the soil [53]. If the final intention of the material is to be used as edible film or coatings, it should be classified as a food additive. Hence, the safety of the materials as additives should be assessed before placing in community market and only ingredients that are listed in authorized substances (EC 1331/208) can be used [54]. According to the EU regulation (EC) NO. 1935/2004 food contact materials should meet the following requirements: (I) Have no danger to human health; (II) does not have a negative impact on the food composition; (III) have no impact on the taste, flavor, or texture of food; and (IV) should be manufactured according to the good manufacturing practice. Most biopolymers used as edible films or coatings are approved to be used in food as a GRAS, except for chitosan and cassia gum that are only allowed in supplements and pet foods, respectively. Moreover, if the edible film is from a protein source that might contain allergens, it should be declared on the label [55].

## 2. Animal Origin Co-Products

Meat products are one of the major sources of dietary proteins, especially in developed countries. Forty percent of total protein consumption worldwide is from animal sources, which is expected to increase by 135% by 2050 [56]. Pork, poultry, beef, and mutton are the most consumed meats worldwide and are mainly served as processed meats, sausages, burgers, and pies [57]. Co-products are the secondary materials that can be obtained during the manufacture of the main products [58,59] and in this review the term animal by-product is avoided as this term has particular relevance under EU regulation [60]. Animal protein, chitin, and fat are the main co-products that can be rendered from livestock, poultry, domestic animals, and aquatic species [61] and generally, one-third to half of each animal live-weight used for meat, milk, eggs, and fibers are considered as co-products [58,59]. While direct consumption is the ideal path for meat co-products, many, along with other wastes generated from meat processing, can lead to disposal and cost issues for the industry. Hence, it is suggested to convert them into value-added products [62,63]. The possible applications of meat processing co-products in various industries and their market overview have been discussed by several authors [56,59,64,65].

Direct consumption of meat co-products is relatively low, mainly due to consumer perception, cultural and traditional practices, religious, ethical restrictions, as well as public health-related issues such as bovine spongiform encephalopathy (BSE) and finally economical barriers. Therefore, these products are often used in pet foods, animal feeds, pharmaceuticals, industrial applications such as glue, plant growth promoter, textile, cosmetic, biopolymers, and water treatments [56]. Utilization of animal co-products not only increases the productivity of meat industry by providing value-added ingredients and products, but also assist in reducing the environmental impact related to any waste generated [66].

Fish and fishery products by supplying more than 17% of total proteins consumed in the world, play an important role in world food security and more than 3.1 billion people obtain at least 20% of their dietary proteins from fish [67]. The global per capita consumption of fish in 2012 was 19.2 kg, and it is projected to increase by 21% in 2025. Among the 158 million tons of fish and aquatic products supplied internationally during 2012, 86% is used directly for human consumption (136 million tons), with the remaining co-products (21.7 million tons) utilized in non-food applications such as fish-meals and fish oil production. The processing of fish and shell fish is concomitant with the immense amount of waste (~75% of total fish weight) that can cause serious environmental, health and economic issues [68]. In Europe alone, this is estimated to be 5.2 million tons per year. However, this waste can be considered as high-quality and low-commercial-value raw material for generating valuable downstream products such as chitin, chitosan, collagen, gelatin, keratin, omega-3 fatty acids, peptides, enzymes, oils, and fishmeal [69].

**Figure 4:**
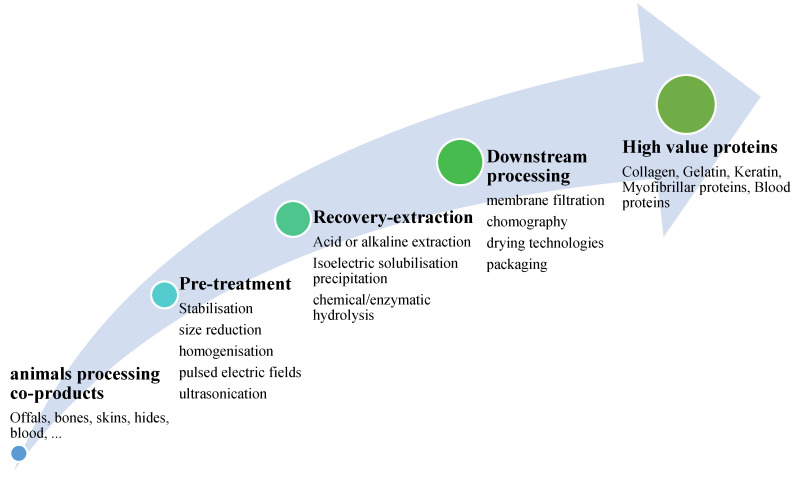
Different stages for protein’s recovery from animals processing co-products and secondary streams [64].

While there are many efforts globally to reduce reliance on animal protein the demand for protein remains, and animal-based protein will continue to be a major player. The wastes and co-products from meat slaughterhouse or processing sectors are rich sources of proteins that can be used as a starter material for producing value-added products [59,63]. Pre-treatment, extraction, and downstream processing are the main steps for recovering proteins from meat processing co-products (Figure 4) [64,70]. Table 1 shows the main proteins that can be obtained from different animal co-products. Studies have shown that animal-based proteins have a high nutritional value, technological and functional properties such as gelling properties, emulsifying, water, and oil-holding capacity [71]. These proteins are natural, cheap, and abundant and due to their considerable functional properties, they can be good candidates for generating biodegradable films. Moreover, protein films are completely compostable and exhibit fertilizing benefits during degradation in soil as they provide a source of nitrogen [36]. Among the proteins obtained from animal sources, collagen, gelatin, keratin, and, myofibrillar proteins have a wide application in biodegradable films production [36].

## 3. Generating Films from Animals’ Proteins

### 3.1. Collagen Films

Collagen is the principle fibrous protein from the skin, bones, ligaments, tendons, and cartilage of bovine animals, pigs, and aquatic species such as fish, jellyfish, and sponges. These fibers provide the structural support to the body organs to ensure the strength and elasticity that is necessary for effective locomotion, tissues regeneration, and repairing through the mechanical and chemical transduction processes [72]. Collagen is generally colourless, opaque, and presents considerable viscoelastic behavior by having a high tensile strength and low elasticity [73]. Collagen molecules are made of three α-chains associated in a triple-helix matrix that are stabilized via intra- and inter-chain hydrogen bonds. This unique conformation leads to continuous repeating sequence of glycine-X-Y amino acids where X and Y are usually proline and hydroxyproline, respectively. Bovine hides (7% of the animal weight) produced during the slaughter process are generally composed of collagen [74]. Other offal such as lungs, tongue, trachea, large blood vessels, or tendons are also rich sources of collagen [59]. Poultry co-products are also considered as an interesting source for processing collagen-based high-value products. Chicken sternal cartilage is a major co-product from poultry processing industry that can be used for extracting collagen type B [75]. Aquatic animals are the other main source of collagen and generally have different functionality than the terrestrial sources. Collagen can be isolated from animal processing co-products via acid-solubilization, pepsin-solubilization, deep eutectic solvent, or supercritical fluid methods [76]. This biopolymer is extensively used in the food industry for its gelling, thickening, and binding characteristics after transforming to gelatin [74]. The global market for collagen in 2016 was around 3.71 billion USD, and it is expected to double by 2025, especially in the beverages, food, cosmetics, and healthcare sectors.

Studies have shown that collagen can be used in biodegradable or edible films manufacturing. Seggiani et al. (2019) investigated the use of hydrolyzed collagen in PBSA (butylene succinate-co-adipate)-based blends and concluded that these blends were of interest in the development of compostable/biodegradable films or molded products. Based on the fertilizing properties of hydrolyzed collagen they suggested potential for use in agricultural applications [77]. Scopel et al. (2016) extracted collagen hydrolysate from chromed leather waste, which is a hazardous waste, and used the collagen for preparing films. The study showed that the addition of glycerol to collagen hydrolysate improved the mechanical properties of the films, and it was comparable to commercial biodegradable films utilized as agricultural mulches [78]. Chen et al. (2017) reported considerable mechanical, water absorption, and biodegradability properties for the collagen scaffolds prepared from Basa fish (*Pangasisus haniltoa*) skin which make it suitable for food packaging application [79]. Ma et al. (2020) prepared blended edible collagen films with sodium alginate/carboxymethyl cellulose in presence of *Lactococcus lactis* viable cells [80] or cell free supernatants of *L. lactis* [81]. These studies showed antimicrobial properties for functional collagen film and their potential use in active food packaging. Some of the recent studies for manufacturing collagen biodegradable films from animal-based co-products are listed in Table 2.

### 3.2. Gelatin Films

Gelatin is a water insoluble protein generated from moderate hydrolysis of collagen (either by chemical, enzymatic, or thermal treatments). The type of collagen, source, and the age of the animal are the key factors affecting the properties of gelatin [93]. The amino acid composition of gelatin is generally made of glycine (27–30%), proline (Pro), and hydroxyproline (Hyp) (20–24%) which closely resembles that of the parent collagen. However, conversion from collagen is accompanied by molecular composition changes in several amino acids. After the partial denaturation of collagen and during the cooling stage, the triple helix structure of gelatin that contains unstructured domains is formed [94]. Gelatin is widely used as a stabilizer, gelling agent, emulsification in food, pharmaceutical, and cosmetic industries [95]. Pig’s skin (46%), bovine hides (29.4%), and beef bones (23.1%) are the major sources of commercial gelatin worldwide. In the past few decades, due to the health concerns attributed to BSE, religious restrictions for using products from pork, environmental, and economic issues related to the fish industry co-products, the interest in extraction of gelatin from marine species has gained much attention. Fish skin, bone, scales, pre-cooked fins, the solid wastes from surimi processing, offal from processing or semi-processing of fish products, farm raised alligator bones, giant Red Sea cucumber are some examples of the potential marine co-products that can be employed as collagen and gelatin sources [93].

Studies have shown that gelatin films have a high mechanical strength, transparency, and barrier properties that make them suitable for food-packaging application [96]. However, the mechanical and barrier properties of gelatin films are highly dependent on the amino acid composition and the molecular weight of the polymer. The composition of amino acids, which differs between species, and the processing conditions affect the molecular weight distribution of the gelatin. According to the United States Food and Drug Administration (FDA), EC, FAO, and WHO, gelatin is a safe additive for the application in the food industry and can be used widely in biodegradable or particularly in edible packaging [95]. Gelatin films due to their high moisture absorbing nature, exhibit a high swelling and dissolving ability when in contact with foodstuff or surfaces contained high amount of moisture [97]. Microbial transglutaminase that is used widely for enhancing the mechanical and barrier properties of gelatin or protein films, is also confirmed as a GRAS (generally recognized as a safe) substance by the FDA since 1998 [98]. Sodium trimetaphosphate (STMP), sodium tripolyphosphate (STP), polyphenols are other safe and non-toxic cross linkers than can be used for improving gelatin or collagen films [99].

Extraction conditions also influence the physical properties of gelatin films. Weng et al. (2014) studied the effect of extraction condition on the final characteristics of gelatin film from fish scale. The results showed that different extraction pH, from 3 to 9, resulted in different amino acid composition, mechanical properties, water barrier, and optical properties of final films [100]. Gelatin film, due to the thermal reversible network and sensitivity to water, has a limited application for long-term packaging purposes. Chemical modification or cross-linking are suggested techniques to improve the mechanical and barrier properties of gelatin films. Fabrication of water-insoluble gelatin film by microbial transglutaminase (MTGase) was carried out by Ma et al. (2020) [101]. These researchers reported that the resulted gelatin could retain its original shape at ambient and boiling water. Gelatin films cross-linked with MTGase had a high molecular weight polymer chains, stable network, and relatively higher mechanical properties compared to control films. Gelatin is one of the most studied biopolymers for active edible packaging. As summarized in Table 3 different studies have evaluated the effect of incorporating essential oils [102,103,104,105,106], probiotic microorganisms [10,107,108], particles reinforcements [109,110,111,112,113,114,115,116,117], or used as a blend with other biopolymers [96,118,119,120,121,122,123] into gelatin films.

### 3.3. Keratin Films

Keratin is a general term for a variety of insoluble structural proteins which make up the main constituents in hair, wool, horns, hooves, and nails. Keratin proteins are rich in sulfur-containing amino acids (cysteine) giving them the ability to form disulfide bonds with other intra and intermolecular cysteine residues. The presence of disulfide and hydrogen cross-links in keratin proteins, give them a crystalline structure, high resilience, and rigidity [128]. Each year around 2.5 million tons of wool and 65 million tons of feathers are produced around the world; of which, wool is used in the textile industry and in small-scale/low-value applications and the rest is disposed through incineration or landfill [129]. The amount of cysteine from wool and feather sources are 11–17%, and 7%, respectively. Keratin from mammalians are categorize in two major groups namely hard keratin and soft keratin, which is based on the structural proteins, their functionality, and regulation. Soft keratins provide a mechanical stretching to the epithelial cells that is mainly due to the loosely packed formation of cytoplasmic intermediate filaments. Hard keratins are responsible for the robust structure of the epidermis that is attributed to the formation of ordered arrays of intermediate filaments into the cysteine-rich proteins [130]. Research has shown that keratin can be used for manufacturing sponges and scaffolds, films, fibers, tissue engineering, and regenerative medicine due to its biodegradable, bio-compatible, and biological properties. Different studies have shown that keratin films are commonly too weak and mixing glycerol in the keratin solution leads to more translucent films with improved mechanical properties [131]. Yin et al. (2013) reported that keratin films, due to their good mechanical properties and also pH-responsive behavior, can be used as an excellent candidate for producing controllable drug-released applications in biomedical fields [132]. Keratin films can be also used as a humidity sensor due to their low cost, porous, and rough surface morphology [133]. The study of the effects of physical and chemical treatments on feather keratin films, by Poole and Church (2015), showed that the drying condition has the greatest influence on the properties of the film. Different chemical and physical treatments such as cross-linking by formaldehyde or glutaraldehyde or soaking the films in isopropyl alcohol or weak acid improved the physical properties of keratin films [134]. Keratin has also been used as biodegradable scaffolds for example in tissue engineering [135] or developing 3D-printing responsive materials [136].

As shown in Table 4, studies have been conducted on manufacturing keratin films from different animal co-products. The study showed that keratin films from sheep wool were transparent, a barrier to UV-rays, and exhibited considerable thermal stability up to 200 °C with no inherent thermal transition. Further thermal cross-linking with glycerol and formaldehyde improved the mechanical properties of the film [137]. Edible cross-linked keratin films from poultry feathers with dialdehyde carboxymethyl cellulose (DCMC) were prepared by Dou et al. (2020). Physical analysis showed excellent transparency and UV-barrier properties for the films. Cross-linking enhanced the water barrier and solubility of the films, while the tensile strength and water solubility reduced [138]. Ding et al. (2020) introduced citric acid as a nontoxic and natural cross-linker to prepare feather keratin nanofibers via electrospinning method. The results showed that using citric acid increased the thermal stability of keratin nanofibers and cross-linking significantly increased the mechanical properties (tensile strength and elongation at break) of the films compared to the non-treated fibers. This study confirmed the possibility of using citric acid as a natural cross-linker to increase the physical properties of keratin nanofibers and their possible use in a variety of applications such as biodegradable packaging [139]. In another study, the effect of 1,8-octanediol as a plasticizer on the reduced and native keratin films from duck feather was evaluated [140]. The results showed that the addition of plasticizer enhanced the mechanical and water resistance of films. Using formaldehyde as a cross-linker reduced the tensile strength of films while the elongation at break improved. However, using formaldehydes due to its toxicity, restricts the application of the films in food packaging and biomedicine.

### 3.4. Myofibrillar Proteins

Muscle proteins based on their position in the skeletal muscle and their solubility in water are divided into sarcoplasmic, stromal, and myofibrillar proteins. Myofibrillar proteins are the major constituent of muscle proteins (60%), and present considerable functional properties such as solubility, emulsifying, and gel-forming ability [154]. These proteins are responsible for texture, yield, and sensory characterization of final meat products. Functional properties of muscular proteins is generally categorized into protein-water, protein-fat, and protein-protein interactions and their application in food industry is mainly influenced by the processing conditions such as ionic strength, pH, temperature, and/or the presence of other ingredients [155]. Myosin is the major protein from myofibrillar proteins (55–60% of myofibrillar protein and 30% of total protein) and it is comprised of heavy spherical and light spiral chains [94]. This protein is fibrous, insoluble in water, and it can be solubilized by altering the pH of the solution in acidic or base conditions followed by removal of stromal proteins via centrifugation. Tsermoula et al. (2019) reported that myofibrillar proteins extracted from porcine and bovine hearts, via alkali or acid solubilization, showed excellent gelling properties, but a higher storage modulus after heating was observed for alkali-treated proteins [156]. Films prepared from myofibrillar proteins are opaque and elastic with the hydrophobic interactions as the main interaction involved [157].

Some of the recent advances involving the preparation of biodegradable films from myofibrillar proteins have been summarized in Table 5. Hamaguchi et al. (2007) prepared flexible and semi-translucent films from Blue marlin muscle proteins. Acidic and alkaline conditions improved the tensile strength of the films, while it showed no effect on the extensibility, barrier, and opacity of the films [158]. Another study showed that using gamma irradiation at a dose of 10 kGy improved the water vapor barrier and mechanical properties of myofibrillar protein from fish-processing wastes, while the higher dose (25 kGy) showed a negative impact on physical properties of the protein film [159]. Vasconcelos da Silva Pereira et al. (2019) studied the effect of processing conditions on the physical properties of myofibrillar proteins from fish co-products. It was observed that films produced at the concentration of 1.13% of protein, 35.96% of plasticizer, and drying temperature of 25.96 °C exhibited a homogenous structure, high transparency, excellent mechanical properties, with a low water vapor permeability enabling its use as a food packaging material [160]. The effect of different fatty acids (stearic, palmitic, and caproic) with sodium lauryl sulfate (SLS) as surfactant on the physical properties of myofibrillar proteins from fish filleting residues was studied in another research [161]. The results revealed that the addition of fatty acids and surfactant improved the flexibility and elongation at the break of the films, while the solubility of the films increased. Treating myofibrillar films with alternating current glow discharged plasma for 2 min increased the elongation at break of films while the tensile strength decreased. Treating the films for longer time (5 min) showed a negative effect on the mechanical properties of the films. Plasma treatment increased the color, opacity, solubility in water, and water permeability of the protein films [162]. In general, there are fewer reports of animal myofibrillar proteins being used for manufacturing of films. This is most likely due to the higher market value of these proteins and other uses such as mechanically deboned meats (MDM).

## 4. Potential Underutilized Animal Origin Proteins for Film Development

### 4.1. Blood Proteins

Blood is one of the major protein-rich co-products from the meat industry which is a good source of essential amino acids or heme iron. The main current industrial application of blood collected from animal slaughterhouses is in pet food, animal feed, bio-fertilizers, biogas generation, and biotechnology with a limited amount used in food applications [169]. Whole blood can be separated sequentially into plasma fraction and red blood cells (RBCs). The primary components of plasma are water and proteins including albumin, fibrinogen, and globulin, along with glucose, minerals, and hormones. A key component of RBCs is hemoglobin (35%), which is rich in heme iron with a high bioavailability. Plasma proteins are tasteless and colorless with excellent foaming, gelling, emulsifying, binding, and oil-holding capacity that make them suitable as a food ingredient. Plasma is used extensively in the food industry as a binder in meat products, egg replacer in bakery, pasta fortifications, fat replacers, or even as a polyphosphate or caseinates alternatives [169]. The application of plasma proteins for manufacturing biodegradable films have been reported by several authors.

The first attempt to prepare films from porcine blood plasma was reported by Nuthong et al. (2009) [170]. The authors studied the effect of plasma protein concentration and glycerol content on some physical properties of prepared films. Glycerol increased the elasticity and transparency while it reduced the moisture barrier properties of the film. Preheating the film-forming solutions or modification of the pH improved the properties of the resultant films. However, the solubility of these films was relatively higher which limits their application in the food industry. In another study by the same authors, different cross-linking agents such as caffeic acid and glyoxal were applied to reduce the water solubility of porcine plasma films. The results showed that the caffeic acid negatively affected the appearance of the films and the application of the glyoxal in food products is limited due to its toxic effect [171]. Samsalee and Sothornvit (2019) investigated the effect of blending different ratios of porcine plasma proteins with chitosan. The findings demonstrated that by addition of chitosan, the transparency, water solubility, and water vapor permeability of the blended films reduced, while the thermal stability and mechanical properties increased [172]. Another study by these researchers elucidated that heating up the porcine plasma protein solutions followed by homogenization improved the mechanical and seal strength of the plasma films [173]. Further blending with chitosan or microencapsulated turmeric oil improved the water resistance and mechanical properties of the resultant films. These active films helped to extend the shelf life of packaged rice grains up to 50 days compared to untreated plasma protein films (40 days). Alvarez-Castillo et al. (2019) developed superabsorbent composite material from porcine plasma protein (PPP) and glycerol through the injection molding process and evaluated the characteristics of prepared material. The study showed that the glycerol decreased both the glass transition temperature of Young’s modulus of these materials. Increase in molding temperature leads to the generation of higher tensile parameters due to the protein gelation followed by cross-linking within the structure of material. The resulting bioplastic displayed considerable superabsorbent properties. However, some of the protein dissolved in water due to the high solubility of PPP [174]. Despite this the authors suggested these bioplastics as an attractive, economic, bio-resource, and biodegradable alternative for current synthetic superabsorbent materials. In another study by the same authors, superabsorbent composite materials from PPP and soy protein isolates (SPI) at different ratios were developed and characterized. The results demonstrated thermosetting properties for PPP with the gelation temperature of ~65 °C, while a thermoplastic behavior was reported for the SPI. PPP exhibited a greater deformability to the bio-composites, but the Young’s modulus reduced by an increase in the PPP/SPI ratio. These authors suggested these bio-composites for developing environmentally friendly products and to replace synthetic acrylic derivatives [175]. Alvarez et al. (2021) successfully prepared transparent, water-insoluble edible films from porcine and bovine serum albumin. The plasma albumin films treated with ethanol exhibited a low solubility in buffer solutions at different pH values and microstructure analysis showed a compact and homogenous matrix. Ethanol treatment of blood serum proteins led to the development of films with more transparency and higher mechanical properties [24].

Hemoglobin (Hb) is a highly water-soluble protein with considerable foaming, emulsifying, and swelling properties. However, the dark color and strong metallic flavor of Hb, restricts its application in the food industry. Separating the heme group from globin is a suggested method to improve the flavor and color characteristics of RBC but the resulting globin is more susceptible to denaturation than the native Hb [169]. RBC has the potential to be used as a fat replacer in meat products due to owning a variety of functional properties such as water solubility, water-holding capacity, swelling, foaming, emulsifying properties, and heat-induced paste formation [176]. Different studies have reported the functional properties for blood proteins which make them suitable to be utilized in higher value applications such as the food products [177]. However, the application of these proteins is mainly limited to feed or food ingredients and their utilization for biomaterials such as films and scaffolds should be considered to expand the industrial application of blood proteins. Using blood proteins as a raw material for producing biodegradable packaging materials, will help to the transform the economy into a more sustainable, circular, and resource-efficient bio-economy, while also reducing the dependency on the petroleum materials. Blood proteins can be used as a whole or separated into plasma and RBC for manufacturing films. It is important to note that blood proteins generally have a high solubility in water due to their globular structure. Therefore, in order to use them for the generation of bioplastics, it is necessary to improve the thermal properties and reduce the water solubility of these proteins. Alvarez et al. (2012) reported properties of isolated PPP could be modified via the milliard reaction to improve the thermal stability and emulsifying capacity and reduce water solubility [178]. Moreover, blood, fats and other residues from the animal co-products, possess a high nitrogen content and high levels of BOD and COD, and hence, have the potential to be used indirectly and as a feed in PHA production [179].

### 4.2. Insects’ Proteins

Insects are an emerging source of high-quality proteins that are consumed by two billion people around the world especially in Africa, Asia, and South America. Over 2100 edible insect species have been reported in literatures [180]. Insects due to their high nutritional value, lower carbon footprint compared to farm animals, lower land and water demand, and high feed conversation efficiency, are considered as an environmentally sustainable source for dietary proteins. Insects can be used directly or processed into pastes, powders, proteins, fats, and chitin to increase their acceptability among consumers. Currently, there are fewer reports published about the functional properties of insect proteins. Functional property analysis of proteins from some edible insects (*Gryllodes sigillatus*, *Schistocerca gregaria*, and *Tenebrio molitor*) showed a considerable water-holding capacity, oil-holding capacity, foaming, and emulsification properties for these proteins. Proteins from *T. molitor* showed the highest water holding capacity (3.95 g/g), and the protein from *S. gregaria* exhibited a high emulsion stability (51.31%) that makes them suitable for food applications [180]. Evaluation of gel-forming ability of different insect proteins (*Tenebrio molitor*, *Zophabas morio*, *Alphitobius diaperinus*, *Acheta domesticus*, and *Blaptica dubia*) revealed that gels at the pH 7 and 10 could be formed at the concentration of 30% *w*/*v*. The gelation temperature for the pH of 7 ranged from 51.2 to 63.2 °C [181]. Although insects are available as a food source in some countries, the direct consumption of them is still very low in the rest of the world which is mainly due to the lack of consumer acceptance in particularly in western cultures. Due to the high economical turnover, and functional properties of insect proteins, they can be used indirectly in food industry as food supplements or as a cheap source of biopolymers for different food or pharmaceutical applications. Recent studies have confirmed the novel processing technologies such as enzymatic cross-linking using transglutaminase [182], extrusion technique [183], enzymatic hydrolysis [184] to improve the functional properties of insect proteins and modify them for novel applications.

## 5. Chitosan

Chitosan is a linear cationic polysaccharide that can be obtained from alkaline deacetylation of chitin. Chitin, the second most common biopolymer available in nature, after cellulose, is the main component of the exoskeletons of arthropods such as crustaceans, crabs, lobsters, shrimps, insects’ skeletons, and fungi. Chitosan is the major part of the seafood waste with a low toxicity, biodegradability, stability, and antimicrobial properties [185]. Chitin content in crustacean shells ranges from 13 to 42%. Shrimp processing leads to substantial waste (40–50%), of which 40% is chitin, clearly indicating a need for finding value-added alternatives for this waste [186]. Chitosan and its derivatives have many applications in the food industry, agriculture, pharmacy, medicine, cosmetics, textile, and paper industries. Studies have also shown that chitosan has considerable antioxidant activity and antimicrobial activity against a wide range of Gram positive, Gram negative bacteria, yeasts, and molds [187]. Chemical and biological methods are the most common methods for extraction of chitin from animal wastes and the extracted chitin converts into chitosan by the deacylation process involving the deletion of acetyl group from the chitin [188]. Biodegradable films from chitosan exhibit a good mechanical, transparency, and antimicrobial properties with a moderate permeability to gasses (CO_2_ and O_2_). Chitosan films are non-thermoplastic as it degrades at temperatures lower than those required for prior melting, therefore, this biopolymer is non-extrudable, and cannot be molded, stretched, or heat sealed which increases production costs and limits applications [189]. Moreover, due to the implication of harsh chemical treatments during the processing method of chitosan films, the industrial scale application of chitosan films is unpractical. Chitosan is one of the most extensively studied biopolymers for manufacturing edible films due to its high transparency, good mechanical and barrier properties, low solubility in water or organic solvents, and antimicrobial properties. Blending different polymers is one of the methods suggested to improve the properties of biodegradable films. Chitosan due to its unique glucosamine structure and owning to a cationic nature has the compatibility to blend with different polysaccharides and proteins to improve the physicochemical properties of final film. Moreover, different plant extracts and oils can be incorporated into chitosan films which improve the surface morphologies, water solubility, and contact angle properties of the film [190]. The recent advances in application of chitosan films focused in food packaging have been reviewed extensively elsewhere [187,190,191,192,193].

## 6. PHA from Animal-Sourced Carbon

Polyhydroxyalkanoate (PHAs) are thermoplastic polyesters with the similar properties to conventional plastics that can be synthesized by numerous microorganisms via fermentation of sugars and under a nutritional stress [21]. Generally, more than 250 types of bacteria are recognized for production of PHAs but only a few of them are approved for industrial production of PHAs [194]. During the nutrients shortage or carbon excess condition, the wild type strains of *Ralstonia eutropha* (H16) and *Alcaligenes Latus* can accumulate polyhydroxybutyrate (the most common PHA biopolymer) up to 90% (*w*/*w*) of their dry weight [195]. The industrial fermentation process of PHAs can be carried out in batch or fed-batch reactors where, after the bacterial cells reach a pre-determined cell mass concentration, the nutrients restriction results in the bacteria storing intracellular PHA and increase in size and weight [46]. PHAs are compostable and can be completely degraded in common environments such as in the soil and sea. The high production cost of PHAs (2.2–5.0 euros per kg) is limiting their application as a bulk packaging material, and so far it is mainly utilized in pharmaceutical and medical applications [196]. Food scraps and animal processing co-products can be used as cheap and abundant carbon sources for the production of PHAs [197]. Another study showed that animal waste fats or tallows can be used as a low-cost carbon source for production of PHAs by *R. eutropha* [195]. The application of waste streams from animal processing industry for utilizing PHA has been reviewed in a study by Koller et al. [198]. The purity assessments of PHAs from feedstock is vital as viral, bacterial, plasmid or genetic material may transfer to the final product: if the final polymer is intended to be used for food or medical application, further washing and sterilization is necessary [45].

## 7. Conclusions

Petroleum-based plastics are the major polymers used in the food packaging sector and increased production in the past few decades has led to growing concerns regarding environmental pollution. In recent years, research and innovation on bio-based polymers has provided solutions to help reduce our dependency on fossil-based packaging films. Identifying suitable feedstock for producing these polymers, takes into consideration a number of factors including the quantity available, inherent characteristics of the feedstock, their ability to form suitable polymers, and the environmental benefits of doing so. Employing materials which are side streams, co-products or even waste products can support the concept of a circular bio-economy and provide more sustainable solutions and optimizing utilization of our natural resources. Taking into account the quantities generated annually, and the increasing demand for biodegradable packaging materials in the food and agriculture sector, the processing or fabrication of animal-based products leads to the generation of many such potential feedstock. While the priority use of these products needs to be used as a food source, this is not always a viable option and hence other avenues to commercialization are required: producing biodegradable, non-petroleum-based plastics is one such avenue.

## Figures and Tables

**Table 1:** Overview of the main proteins in some animal co-products.

Co-Product	Main Protein	Protein (%)	Extraction	Functionality	Application
**Blood**	Plasma proteins (albumin, fibrinogen, and globulin) and red blood cells (hemoglobin)	18.5	Using evaporators for producing whole blood. Blood can be also treated with anticoagulant agents and then separated into plasma proteins and red blood cells via centrifugation.	Emulsifying, stabilizer, clarifier, color additive, water and fat binder	Blood sausages, blood pudding, biscuits, and bread
**Heart, liver, lungs, tongue, spleen, meat residues from canning**	Myofibrillar proteins	Heart (17) Liver (19) Lung (15) Spleen (17.9) Tongue (16.3)	Using denaturing solutions containing urea, thiourea, reducing agents such as dithiothreitol and beta-mercaptoethanol, detergents such as sodium dodecyl sulfate, and salts. Acid or alkaline solubilization followed by isoelectric precipitation method. Surimi processing which is based on washing the minced meat to remove the water-soluble proteins, enzymes, blood, and minerals that lead to higher concentration of myofibrillar proteins. In order to increase the protein stability, the product is mixed with a cryoprotectant.	Viscosity and creaminess, Mild water binding and good cooking yield, Emulsifying and foaming	Braised, cooked in liquid, luncheon meat, patty, loaf, broiled, fried, in loaf, patty and sausage, Blood preparations, pet food, Fried, in pies, Cooked in liquid, cured, sausage casing,
**Feathers, hair, wools, horns and hoofs, nails,**	Keratin	Feathers (82) Wool (95) Horns (93.3)	Chemical methods (oxidation, reduction, and hydrolysis), microbial and enzymatic treatment, supercritical water and steam explosion, and microwave irradiation are the main extraction methods for keratin. During the oxidizing method the disulfide bonds oxidize into sulfonic acid using an oxidizing agent. Therefore, a hydrophilic keratin is produced that dissolves the hair. In reducing method micelles in surfactants are used to protect the keratin and avoid oxidation and precipitation during the process. This method leads into keratin with a higher Mw and dissolubility. In hydrolysis method a strong base (sodium hydroxide) is used to break down the disulfide bonds between molecules and decompose the peptide chain which leads to polypeptides with low Mw.	Film forming ability, scaffolds and hydrogels, drug release application	Food industry, cosmetics, biomedical application, textile, biofertilizing
**Hides, skins, Bones, fish cartilage, fish scales, ears**	Collagen	Hide and skins (30–35) Bones (50) Fish scales (41–81) Ear (22.3)	Preliminary washing, cleaning, and separation of the animal parts, size reduction by mincing or cutting samples. Mild chemical pre-treatment to increase the efficiency of the extraction and eliminate non-collagenous materials Alkali or acid treatment to break down the cross-linked collagen before the extraction as collagen exist in cross-linked form in the connective tissue of animals.	Gelling, water and fat binding, emulsifying, and stabilizer	Athletic equipment, reformed sausage casing and cosmetic products, sausage skins, edible gelatin and glue Jelly, pickled, cooked in liquid, boiled, fried

**Table 2:** Collagen films developed from different animal by-product sources.

Source	Aims	Plasticizers	Method for Film Production	Results	Reference
**Hidrogel^®^ B50**	Developing edible films from hydrolyzed collagen, sucrose, and cocoa butter	Sucrose	Solvent casting	Sucrose and cocoa butter reduced the TS of films. Plasticizer improved the elongation of the films. Sucrose increased transparency of films while cocoa butter had negative effect on it. Films contain above 17.5% of hydrolyzed collagen had more homogenous surfaces	[82]
**Bovine hides**	Manufacturing collagen films incorporated with laponite^®^ nanoparticles	Glycerol	Casting	Laponite significantly enhanced the surface roughness of the films while other parameters such as thickness, moisture content, gloss, color, transparency, mechanical, and barrier properties remained intact. Nano-bio-composite films showed a lower melting enthalpy than pure collagen films.	[83]
**Fish skin**	Scaling up of collagen and sodium alginate blended films	Glycerol	Casting machine	The addition of sodium alginate enhanced the viscosity, thermal stability, and TS of collagen films, while the elongation, and WVP remained unchanged. Films made from collagen: sodium alginate 10:2 showed the best rheological and physical properties. Collagen/sodium alginate films were successfully scaled up.	[84]
**Bligon Goatskin**	Producing edible films from collagen extracts and glycerol	Glycerol	Solvent casting	Different concentrations of plasticizer, significantly affected the thickness, tensile strength, and elongation of films but had no effect on solubility, WVTR, and water activity of films. Film contain 80% of glycerol (based on collagen) showed the best mechanical and physical properties.	[85]
**Bovine connective tissue**	Effect on different cross-linkers on the barrier properties of collagen films	Lecithin	Solvent casting for chemically modified films and extrusion for thermally modified films	Thermal cross-linking significantly improved the water resistance of collagen film (up to 70% after 2 h at 80 °C). However, chemical cross-linking with glutaraldehyde, glyoxal, and/or formaldehyde (10% *w*/*w* of collagen dry matter) leads to highest water resistance (100% after 2 h at 80 °C). Chemical cross linking reduced the degradation rate of films (90% degradability at 58 °C during 38 days).	[86]
**Cow’s hide**	Effect of apatite reinforcement on physical properties of collagen film	Glycerol	Solvent casting	Apatite particles presented in surface of the film and also increased the compactness of inner side of films with less porous compared to pure collagen film. Incorporation of Apatite significantly enhanced the TS and reduced WVP of films. Apatite decreased the solubility and enhanced the thermal stability of collagen fiber films.	[87]
**Tilapia skin collagen**	Developing blended collagen films with Pachyrhizus starch or rambutan peel phenolics	Glycerol	Solvent casting	The addition of starch and phenolics significantly increased the opacity and thickness of films while water solubility, EAB, and WVP reduced. Highest TS observed for collagen film loaded with 10% starch and 0.5% phenolics. Thermal stability of collagen improved by modification of films and SEM analysis showed a more smooth, uniform, and dense surface for composite films.	[88]
**Trimmed skin waste from leather industry**	Producing blended films from collagen, starch, and soy protein	-	Solvent casting	TS of collagen films increased as the concentration of starch increased, while EAB of films increased by the increase in soy protein in formulation. Hybrid films showed moderately higher thermal stability. SEM images revealed smoother surface for starch-loaded films; soy protein increased the roughness. Hybrid films showed an increase in swelling and in vitro biodegradation compared to pure collagen films.	[89]
**-**	Developing blended films from collagen, methylcellulose, and whey protein	Glycerol	Solvent casting	Collagen films showed the highest EAB (101.4%) and addition of methylcellulose improved technological properties of films such as TS, barrier, and thermal properties of collagen and whey protein films.	[90]
**Bovine hides**	Preparation collagen-2 hydroxyethyl cellulose hybrid films	-	Solvent casting	Cross-linking with cellulose derivatives improved the TS of dry collagen films (22 to 58.9 MPa) compared to pure collagen. Hydrated films showed lower TS and higher EAB compared to dry hybrid films. Cross-linking improved the thermal stability of films. The presence of cellulose improved the bio-stability and biocompatibility of the films with a controlled degradation compared to pure collagen film.	[91]
**Bovine skin splits**	Manufacturing collagen films incoprorated with carboxylated cellulose nanofibers (CNF)	Glycerol	Solvent casting	CNF increased the collagen fibers suspensions and TS of collagen films while EAB reduced. WVP and oxygen permeability of CNF loaded films significantly improved. Microstructure analysis showed that CNF homogenously embedded into collagen fiber matrix and increased the thickness, opacity, and swelling of films.	[92]

**Table 3:** Gelatin films developed from different animal by-product sources.

Source	Extraction Method	Aims	Plasticizers	Method for Film Production	Results	Reference
**Fish skin (Cynoscion acoupa)**	Acidic extraction	Study the effect of palm oil and essential oils on physical properties of gelatin film	Glycerol, palm oil, and gum Arabic as surfactant	Solvent casting	Addition of palm oil increased the elasticity and thickness of the films. Incorporation of clove oil into the gelatin films increased the antimicrobial activity.	[121]
**Cuttlefish (*S. officinalis*) by-products**	Alkali extraction by NaoH	Manufacturing gelatin films loaded with enzymatic protein hydrolysates	Glycerol	Solvent casting	Applying cuttlefish skin protein isolates and hydrolysates into gelatin film increased the UV-barrier properties and glass transition temperature of the film while the EAB and TS significantly reduced. Protein isolates increased the antioxidant activity of gelatin film	[124]
**Chicken skins**	Alkali extraction with NaOH	Preparation rice flour blended gelatin films	Glycerol	Solvent casting	The addition of rice flour increased the WVP of films while the solubility decreased. Rice flour decreased UV and light transmission and improved the thermal properties. Blended films showed improvement in TS and EAB and addition of rice flour at 20% (*w*/*w*) showed the best results.	[122]
**Tilapia fish (*Oreochromis niloticus*) skins**	Alkali extraction followed by acidic treatment	Replacing glycerol with fatty acid sucrose esters (FASEs) in gelatin films	Glycerol and fatty acid sucrose esters (FASEs)	Solvent casting	Replacing glycerol with FASEs reduced the WVP of the gelatin films but increased the opacity and water solubility of films. FASEs increased the TS and YM while the EAB decreased. Gelatin-PASE films showed a rougher surface compared to control gelatin-glycerol films	[125]
**Tilapia scale gelatin**	-	The effect of electron beam irradiation (EBI) and antioxidants from bamboo leaves (AOB) on gelatin films	Glycerol	Solvent casting	The results showed that EBI and AOB improved the TS, denaturation temperature, opacity, and microstructure of gelatin films. EBI at the dosage of 5 and 7 kGy showed the highest mechanical and thermal properties but the WVP increased. Irradiation contributed in cross linking between gelatin and AOB.	[104]
**The bones of red snapper (Rs) (*Lutjanus campechanus*), and** **grouper (Gr) (*Epinephelus chlorostigma*)**	Alkali extraction followed by acidic treatment	Develop composite films from nanoclay, montmorillonite (MMT), and chitosan in gelatin films	Sorbitol	Solvent casting	Gelatin films from grouper bone exhibited the highest TS and YM and the highest EAB was observed for red snapper films. Addition of chitosan and MMT improved the TS and barrier properties of films.	[126]
**Hagfish skin**	Alkali extraction by NaOH	Developing gelatin films loaded with cinnamon-bark essential oil (CBO)	Fructose, glycerol, and sorbitol	Solvent casting	Addition of CBO to the gelatin films up to 1%, decreased the TS while the EAB of the films improved. CBO increased the hydrophobicity of the film’s surface and exhibited antimicrobial and antioxidant activities.	[105]
**skin of grey triggerfish (*Balistes capriscus*)**	Alkaline extraction followed by acidic treatment	Developing antimicrobial gelatin films enriched with orange peel pectin	Glycerol	Solvent casting	Blending gelatin with pectin reduced the wettability of the gelatin film. Film prepared from equal ratios of gelatin and pectin showed the highest glass transition temperature and TS. Blended films showed antioxidant and antibacterial properties that helped to improve the physicochemical, textural, and microbial stability of wrapped cheese during storage.	[123]
**Tilapia scales**	Alkaline extraction followed by acidic treatment	Formulation of gelatin films with anthocyanin nano-complexes	Glycerol	Solvent casting	Studying the gelatin extracted from different pH 3 to 9 showed that the films with highest TS prepared from gelatin were extracted at pH of 5 and film strength decreased by lowering the extraction pH. The extraction pH had no effect on WVP, color, and transparency of films. α-helix showed the highest influence on the formation of films compared to the molecular weight and Tg.	[100]
**Chicken’s skin**	Acid–alkaline pre-treatment	Optimization of gelatin films from chicken skin and different amount of plasticizer	Glycerol	Casting technique	The optimization process for production of gelatin films at different concentration of gelatin and glycerol showed that at the concentration of 4 g for gelatin and 1.5 g of glycerol, the best mechanical (TS of 3.81 N/mm and EAB of 3.04%) and barrier properties (WVP of 1.27 × 10^−9^ kPa) were observed	[127]
**Bovine bones**	Acid treatment	Developing water insoluble cross-linked gelatin films	-	Solvent casting	Using microbial transglutaminase significantly increased the molecular weight, stabilized network structure, and improved the mechanical properties, and the final films were water insoluble.	[101]
**Dried Alaska pollock by-product**	Alkali treatment	Developing gelatin films and coatings loaded with rosewood essential oil (RO) and pine needle extract (PE)	Fructose	Solvent casting	Incorporation of RO and PE in gelatin film increased the antioxidant and antimicrobial activities. Films loaded with 1% PE showed more desirable physical properties and helped to reduce the total aerobic bacteria, yeast, and molds, and also reduced the weight loss and anthocyanin changes in stored grape berries.	[106]

**Table 4:** Keratin films developed from different animal by-product sources.

Source	Extraction Method	Aims	Plasticizers	Method for Film Production	Results	Reference
**Chicken feathers**	Alkaline hydrolysis	Producing bioplastic from keratin and microcrystalline cellulose	Glycerol	Solvent casting	Keratin was extracted from chicken feathers using sodium sulfide and used for producing biodegradable films. Prepared films showed a TS of 3.62 MPa, YM of 1.52 MPa, and EAB of 15.8% that makes it suitable for producing bioplastic films	[141]
**Sheep Wool Keratin**	Alkaline mild oxidative method	Cross-linking of sheep wool keratin with sodium dodecyl sulphonic acid (SDS)	Glycerol	Solvent casting	Prepared films showed considerable transparency, UV-barrier properties, and thermal stability up to 200 °C. Using SDS leads to more hydrophobic material but with less plasticizing effects. Cross-linking of films with formaldehyde leads to high mechanical strength. Biodegradability assay showed 40% of degradation for films after 5 days of composting.	[137]
**Chicken feather**	Hydrolase feather using urea, Na_2_S.9H_2_Oand SDS	Developing keratin films loaded with dialdehyde carboxymethyl cellulose (DCMC)	Glycerol	Solvent casting	Covalent and hydrogen bonds occurred between keratin and DCMC. Cross-linked films showed good UV-barrier properties and transparency. However, DCMC decreased the TS and moisture sensitivity of the films compared to the control keratin films.	[138]
**Chicken feather**	Acylation process	Producing thermoplastic films by acylation of keratin	Glycerol	compression-molding	Acylation process leads to develop thermoplastic keratin as a green and inexpensive product. Acylated keratin showed the melting peak around 115 °C which was slightly higher than weight loss and thermal degradation. Produced films were transparent and biodegradable.	[142]
**Chicken feather**	Sulphitolysis method	Studying the effect of processing condition and blending keratin on the films	PLA nanofibers	electrospinning	The extracted keratin exhibited a non-Newtonian behavior that could not form nanofiber via electrospinning. Therefore, by blending with PLA (10% wt), the keratin-based material could be prepared. PLA decreased the glass transition temperature of keratin.	[143]
**Bovine hair**	Immunization via sodium hydroxide	Using hair wastes as keratin source for films production	Glycerol, lactic acid	Thermo-compression	Films prepared by thermos-compression (147 kN, 120 °C or 160 °C, and 4 min). The films were opaque/dark and higher processing temperature or lactic acid led into a higher solubility in water. By increasing the amount of plasticizers, the amount of TS, YM, and EAB of the films decreased while the strain at break increased.	[144]
**Chicken feather**	Extract by peracetic acid Solution followed by centrifuge	Producing keratin films by electrospinning and citric acid vapor modification		electrospinning and citric acid (CA) vapor modification	CA vapor cross-linking increased the nanofibers diameter compared to water vapor. CA significantly improved the thermal stability and water resistance of keratin nanofibers. TS and EAB for CA cross-linked keratin improved 1.2 and 2 times compared to untreated nanofibers. CA vapor treatment increased the hydrophobicity of nanofibers.	[139]
**Duck feathers**	Solution containing urea, SDS, and sodium bisulfite	Study the plasticizing effect of 1,8-Octanediol in keratin films	1,8-Octanediol (OD)	Solvent casting	Two types of keratin were extracted (reduced and native keratin). The presence of OD increased the hardness of films. Cross-linking with formaldehyde improved the mechanical properties and water resistance of the films.	[140]
**Chicken feathers**	Alkaline agent (NaOH)	Improve properties of keratin films by using microcrystalline cellulose	PVA/glycerol	Solvent casting	Addition of microcrystalline cellulose (2%) increased the hydrogen bonds between keratin protein and cellulose. MC improved the surface morphology and increased the crystallinity and thermal properties of keratin film.	[145]
**White chicken feathers**	NaOH solution followed by centrifuge	Manufacturing blended keratin films incorporated with essential oils	Sorbitol	Solvent casting	Addition of gelatin significantly increased the TS and EAB of keratin films. Further addition of cinnamaldehyde improved the mechanical properties of composite films. Composite films loaded with clove oil used for packaging smoked salmons and the results showed that it decreased the population of pathogenic microorganisms during storage of salmon and it also reduced the peroxide value and thiobarbituric acid compared to control samples.	[146]
**Chicken feathers**	NaOH solution followed by centrifuge	Study the effect of nanoclays and plasticizers on keratin films	Glycerol: Sorbitol	Solvent casting	The use of 1:3 or 0:1 (*w*/*w*) blend of glycerol and sorbitol showed the best mechanical properties for the films. However, the incorporation of nanoclay improved the physical properties of keratin films by increasing TS and decreasing WVP compared to pure keratin films. Films incorporated with 3% of nanoclay showed the most suitable mechanical and barrier properties.	[147]
**Chicken feathers of *Gallus gallus domesticus***	Sodium bisulfite, urea, and SDS solution	Developing keratin-alginate fibers for industrial biodegradable materials	Glycerol	Solvent casting	Dual cross linked keratin-alginate fibers were successfully produced. N-(3-Dimethylaminopropyl)-N0-ethylcarbodiimide hydrochloride and calcium ions were the first and second cross-linking agents, respectively. Cross-linking significantly improved the strength, modulus, and toughness by 27, 20, and 33%, respectively. Cross linking improved the gravimetric toughness of the fibers and the authors suggested that for textile or tissue engineering applications.	[148]
**Goat hoof**	Soxhlet apparatus	Biopolymer film fabrication from keratin, fibrin, and gelatin	2% (*w*/*w*) glycerol and 3% (*w*/*w*) tetrathylorthosilicate	Solvent casting	Wound-healing films from keratin, blood fibrin, gelatin, along with mupirocin were successfully prepared. The study showed biompactibility, cell viability, cell adhesion, and proliferation of blended polymers which can be used as a cheap and biodegradable film for supporting wound healing.	[149]
**Chicken feather**	Sodium bisulfite, urea, and SDS solution	Manufacturing keratin films cross-linked by dialdehyde starch (DAS)	Glycerol	Casting method	Cross-linking increased the compactness, amorphous structure, and transparency of keratin films. Cross-linked films showed lower solubility and the films with 2% DAS had a higher EAB and WVP compared to control films while the TS decreased.	[150]
**Chicken feather**	Alkali extraction and acid precipitation by urea and sodium sulfide	Developing bioplastics from hydrolyzed keratin films	Glycerol	Hot-pressing process	The results showed that high temperature and pressure improved the compatibility between glycerol and hydrolyzed keratin molecules. By increasing the glycerol content in films, the TS decreased while the solubility and EAB increased. Prepared films exhibited a low amount of solubility in water and addition of higher amount of glycerol increased WVP of films.	[151]
**Quail feathers**	Alkali extraction by NaOH and sodium sulfide	Manufacturing antibacterial keratin scaffolds incorporated with silver nanoparticles	-	Blending with PVA as a host polymer	Scaffolds with 0.75 wt% of keratin produced more uniform structure with less beads formation and exhibited a high antibacterial activity against Gram-positive (99.9%) and Gram-negative (98%) bacteria. Presence of keratin and silver nanoparticles, reduced the cytotoxicity and enhanced the viability of scaffolds.	[152]
**Chicken feathers**	Using urea, SDS, 2-mercaptoethanol, and tris(hydroxymethyl)-aminomethane solution	Study the effect of polyethylene glycol molecular weight on physical properties of films	Polyethylene glycol with different molecular weights (400, 1500, 4000, 6000)	Solvent casting	By increase in PEG molecular weight, the equilibrium moisture of keratin films reduced. PEG 400 was the best plasticizer in term of lower water solubility and WVP and also reduced the brittleness of the films.	[153]

**Table 5:** Myofibrillar protein films developed from different animal by-product sources.

Source	Extraction Method	Aim	Plasticizers	Method for Film Production	Results	Reference
***Pangasius* (Basa) fish waste**	Sodium chloride solution	Using gamma irradiation to modify myofibrillar films	Glycerol	Solvent casting	Non-irradiated films showed the lowest TS and the highest EAB. Irradiation increased the yellowness of the films. Films irradiated with 10 KGy exhibited the highest WVP and lowest water solubility.	[159]
**Tilapia (*Oreochromis niloticus*) waste**	Sodium chloride solution	The effect of plasticizers on physical properties of films	Glycerol, Sorbitol, and polyethylene glycol	Solvent casting	Glycerol plasticized film showed the highest EAB and WVP. The highest TS and water solubility were observed for sorbitol-containing films. Films plasticized with PEG exhibited a yellowish color and low light transmittance at 280 nm.	[163]
**Byproducts of Gilded catfish** **(*Brachyplatystoma rousseauxii*)**		Optimization of processing parameters to produce films from fish by-products	Glycerin	Casting method	Processing optimization using a central composite rotatable design showed that at the protein concentration of 0.79% *w*/*v* and 40% *w*/*w* of plasticizers, a homogenous and transparent bioplastic is obtained that shows excellent mechanical and barrier properties.	[69]
**Filleting residues of gilthead bream (*Brachyplatystoma roussauxii*)**	Sodium chloride solution	Blended myofibrillar protein film with chitosan	Glycerol	Casting method	Films made from 1.3% (*w*/*v*) myofibrillar proteins, 30% (*w*/*w*) of chitosan, and 40% (*w*/*w*) of glycerol was the optimal formulation. Chitosan increased WVP of the films while it improved the mechanical properties, solubility, swelling, UV-barrier, and thermal stability compared to the control films.	[164]
**Filleting scrapings and skin of king weakfish (*Macrodon ancylodon*) fillet**	phosphoric acid treatment followed by sodium chloride solution	Developing mixture films of gelatin and myofibrillar proteins	Glycerol	Casting method	Myofibrillar films showed a high TS and less flexibility compared to gelatin film. Films prepared from mixing myofibrillar protein and gelatin exhibited the lowest WVP, water solubility, and higher transparency with improved mechanical properties.	[165]
**Acoupa weakfish** **(*Cynoscion acoupa*) fillet residues**	Metaphosphoric acid (HPO_3_) solution treatment followed by sodium chloride solution extraction	Myofibrillar protein films added with fatty acids and surfactants	Fatty acids (stearic, palmitic, and caproic), surfactant (SLS), and glycerol	Solvent casting	Addition of surfactant and fatty acids increased the elongation of the films compared to control film. Fatty acids increased the solubility of film up to 100% and decreased the transparency of film. Films contain 5% stearic acid and 10% SLS with 10% palmitic acid and 20% SLS exhibited the highest TS.	[161]
**Acoupa weakfish** **(*Cynoscion acoupa*) filleting residues**	Using sodium chloride solution	Optimization of myofibrillar films from fish by-product	Glycerol	Solvent casting	The optimization process of preparing biodegradable using placket-Burman Fractional Design revealed that ideal condition to produce films was at the concentration of 1.13% *w*/*v* protein, and 35.96% *w*/*w* of plasticizer at 25.96 °C of drying temperature that lead in a more homogenous and transparent film with considerable TS, flexibility, and water barrier properties	[160]
**Crayfish flour**		Studying the effect of sodium sulfite and urea on physical properties of film	Glycerol	Injection molding	The processing ability of myofibrillar films increased after the addition of sodium sulfite as the reducing agent or urea as the denaturing agent. However, the effect of these agents on the properties of the films were unclear. Mechanical properties of myofibrillar protein films were lower than albumin protein isolate films.	[166]
**Tilapia fish**	Sodium chloride solution	Fabrication of myofibrillar films containing catechin and Kradon extracts	Glycerol	Solvent casting	By increasing catechins concentration in film, the EAB and WVP decreased while the brightness enhanced. Addition of both extracts increased the UV-barrier and thermal stability of films. The presence of extracts increased antioxidant activity of films but showed no effect on antimicrobial activity.	[167]
**Whitemouth Croaker muscle wastes**	Sodium chloride solution	Comparing the films prepared from fish’s residues (protein isolate film) and from muscle residue (myofibrillar films)	Glycerol	Casting method	The results showed that films from protein isolates exhibited a higher solubility in water. Films prepared from myofibrillar protein showed a higher TS and lower amount of WVP in different protein concentrations.	[168]

## Data Availability

All supporting data are reported in the manuscript.
